# Reply to ‘Climate of doubt: a re-evaluation of Büntgen and Di Cosmo’s environmental hypothesis for the Mongol withdrawal from Hungary, 1242 CE’

**DOI:** 10.1038/s41598-017-12126-8

**Published:** 2017-10-05

**Authors:** Ulf Büntgen, Nicola Di Cosmo

**Affiliations:** 1 Department of Geography, University of Cambridge, Cambridge, CB2 3EN UK; 2 Swiss Federal Research Institute WSL, 8903 Birmensdorf, Switzerland; 3 Global Change Research Centre and Masaryk University, 613 00 Brno, Czech Republic; 4 Institute for Advanced Study, School of Historical Studies, 1 Einstein Dr, Princeton, NJ 08540 USA; 5 Princeton University, Department of East Asian Studies, Princeton, NJ 08540 USA

## Abstract

In our 2016 article in *Scientific Reports*, we advanced a new hypothesis for the Mongol withdrawal from Hungary in 1242 CE, based on a joint analysis of climatic, environmental, and historical data. The re-evaluation now offered by Pinke *et al*. casts doubt on this hypothesis. However, their arguments are based on a level of generality that fails to appreciate the specific conditions of the Mongol invasion, do not offer new or different climatic data, and are supported by anachronistic production data and environmental information, which cannot be related to the period in question. While we acknowledge the importance of an open debate, we stand by our conclusions.

## Introduction

Pinke *et al*.^1^ (P) argue against consideration of climatic factors in the 1242 CE sudden withdrawal of the Mongols from Hungary. While acknowledging their contribution to the long-lasting debate, we dispute each of their five key arguments^1–5^. Moreover, we re-emphasize the importance of multifaceted environmental aspects and the importance of annually resolved, absolutely dated and spatially explicit natural archives and historical sources^2^, when linking climate variability with human history^3^.

P contend that:

1. a letter by King Bèla to the Pope (1250 CE) asserts that, had the Mongols attacked Hungary again, they could have settled there. This political document, however, bears no relation to the condition of Hungary in spring 1242. The Mongols might have settled in Hungary under normal conditions, and indeed preparations were made in 1241. Flooding and swamping, however, possibly hampered their military operations, and made the Mongols weary of settling.
2. precipitation surplus favors agricultural production. We agree that this might be true in a steppe environment, but we need to consider the specific conditions of 13th-century Hungary. Climatic changes in the late 12th and early 13th century most likely raised the water level, while an overall high groundwater table contributed to the shrinkage of areas suitable for occupation and arable land^4^. Because of rising water levels in the 12th century, villages were often relocated to higher elevation^5^. From the 18th century onwards, agricultural productivity in Hungary was transformed due to extensive drainage, turning marshland into plough land^6^. Data used by P are for 1921–2010^7^, and therefore anachronistic. P also confuse military capabilities with economic potential. On the contrary, we argue that wetter conditions hampered mobility of the Mongol cavalry, and thus critically contributed to their low success rate in 1242 and subsequent withdrawal.
3. climate did not contribute to Hungary’s famine in 1243. Recent research, however, posited environmental impacts on the 1243 crisis^8^. Furthermore, Vadas^9^ attributes the famine of 1315–17 in Hungary to lower temperatures and flooding, which supports our contention that famines in the 13th and 14th centuries can be associated with flooding extremes and generally colder temperatures.
4. most of the destruction occurred in the central Hungarian plain. Indeed, the Mongol invasion of 1241 was highly successful. However, in the following year, the Mongols met different conditions and had to withdraw; western Hungary thus came out largely unscathed. The different impact of the Mongol conquest in these two regions should take into account how different weather conditions affected military operations.
5. a painting from Székesfehérvár provides useful insights. Nevertheless, the picture is anachronistic and does not illustrate conditions in spring 1242. That Székesfehérvár was surrounded by marshes “to inhibit the advance of a hostile army” actually supports our point that marshes would have hindered the Mongol army.

Finally, we have to stress the need for overcoming deterministic and reductionist tendencies, as well as for using truly cross-disciplinary approaches between natural sciences and the humanities when interpreting archaeological remains and historical events in the context of past climatic changes^10,11^.
