# Publisher Correction: Reply to ‘Climate of doubt: a re-evaluation of Büntgen and Di Cosmo’s environmental hypothesis for the Mongol withdrawal from Hungary, 1242 CE’

**DOI:** 10.1038/s41598-018-25733-w

**Published:** 2018-05-10

**Authors:** Ulf Büntgen, Nicola Di Cosmo

**Affiliations:** 1 Department of Geography, University of Cambridge, Cambridge, CB2 3EN UK; 2 Swiss Federal Research Institute WSL, 8903 Birmensdorf, Switzerland; 3 Global Change Research Centre and Masaryk University, 613 00 Brno, Czech Republic; 4 Institute for Advanced Study, School of Historical Studies, 1 Einstein Dr, Princeton, NJ 08540 USA; 5 Princeton University, Department of East Asian Studies, Princeton, NJ 08540 USA

Correction to: *Scientific Report*s 10.1038/s41598-017-12126-8, published online 05 October 2017

In this Article, the DOI provided in Reference 1 is incorrect. The correct Reference 1 appears below.
